# Increased Sensitivity of Mice Lacking Extrasynaptic δ-Containing GABA_A_ Receptors to Histamine Receptor 3 Antagonists

**DOI:** 10.3389/fphar.2020.00594

**Published:** 2020-05-06

**Authors:** Shamsiiat Abdurakhmanova, Milo Grotell, Jenna Kauhanen, Anni-Maija Linden, Esa R. Korpi, Pertti Panula

**Affiliations:** ^1^Department of Anatomy, Faculty of Medicine, University of Helsinki, Helsinki, Finland; ^2^Department of Pharmacology, Faculty of Medicine, University of Helsinki, Helsinki, Finland

**Keywords:** extrasynaptic GABAA receptor, GABAA δ subunit, Gabrd KO mice, Electroencephalogram, histamine, GABA, ciproxifan, pitolisant

## Abstract

Histamine/gamma-aminobutyric acid (GABA) neurons of posterior hypothalamus send wide projections to many brain areas and participate in stabilizing the wake state. Recent research has suggested that GABA released from the histamine/GABA neurons acts on extrasynaptic GABA_A_ receptors and balances the excitatory effect of histamine. In the current study, we show the presence of vesicular GABA transporter mRNA in a majority of quantified hypothalamic histaminergic neurons, which suggest vesicular release of GABA. As histamine/GABA neurons form conventional synapses infrequently, it is possible that GABA released from these neurons diffuses to target areas by volume transmission and acts on extrasynaptic GABA receptors. To investigate this hypothesis, mice lacking extrasynaptic GABA_A_ receptor δ subunit (Gabrd KO) were used. A pharmacological approach was employed to activate histamine/GABA neurons and induce histamine and presumably, GABA, release. Control and Gabrd KO mice were treated with histamine receptor 3 (Hrh3) inverse agonists ciproxifan and pitolisant, which block Hrh3 autoreceptors on histamine/GABA neurons and histamine-dependently promote wakefulness. Low doses of ciproxifan (1 mg/kg) and pitolisant (5 mg/kg) reduced locomotion in Gabrd KO, but not in WT mice. EEG recording showed that Gabrd KO mice were also more sensitive to the wake-promoting effect of ciproxifan (3 mg/kg) than control mice. Low frequency delta waves, associated with NREM sleep, were significantly suppressed in Gabrd KO mice compared with the WT group. Ciproxifan-induced wakefulness was blocked by histamine synthesis inhibitor α-fluoromethylhistidine (αFMH). The findings indicate that both histamine and GABA, released from histamine/GABA neurons, are involved in regulation of brain arousal states and δ-containing subunit GABA_A_ receptors are involved in mediating GABA response.

## Introduction

Hypothalamic tuberomammillary histaminergic neurons form a relatively small population of cells involved in the regulation of wakefulness, energy homeostasis, appetite, reward, and cognition (Brown et al., 2001; Haas and Panula, 2003; Panula and Nuutinen, 2013). Release of neuronal histamine is strongly correlated with the wake vigilant state and regulated by a variety of sleep- and wake- promoting neuron systems (Brown et al., 2001; Zant et al., 2012; Rozov et al., 2014; Saito et al., 2018).

Hypothalamic histaminergic neurons contain multiple other neurotransmitters and peptides (Airaksinen et al., 1992; Sundvik and Panula, 2012). GABA was reported to be present in all (Airaksinen et al., 1992) or a majority (Trottier et al., 2002; Sundvik and Panula, 2012; Yu et al., 2015) of histaminergic neurons, depending on detection method used and species studied. Co-existence of GABA and histamine in hypothalamic neurons has been known for a long time (Takeda et al., 1984), but the role of GABA has been poorly understood.

Although GABA could be released non-canonically *via* vesicular monoamine transporter (Vmat2) (Tritsch et al., 2012), in histaminergic cultured neurons, GABA is not co-localized with either histamine or Vmat2 immunoreactivity (Kukko-Lukjanov and Panula, 2003). The presence of vesicular GABA transporter (Vgat) in histaminergic neurons [identified by presence of histidine decarboxylase (Hdc)] is controversial: while a co-localization study using transgenic reporter mice and immunostaining shows that the majority of Hdc-positive cells also contain Vgat (Yu et al., 2015), another *in situ* hybridization and immunostaining study suggests that only 7% of Hdc immunoreactive cells contain *Vgat* mRNA (Venner et al., 2019). To clarify this controversy, we used double fluorescence *in situ* hybridization (dFISH) and quantified the number of histaminergic neurons expressing *Gad67* and *Vgat* mRNA, which encode GABA-synthesizing enzyme glutamic acid decarboxylase 67 (Gad67) and Vgat.

Currently, it is assumed that GABA from histamine/GABA neurons diffuses by volume transmission to many brain regions and provides tonic inhibition by acting on extrasynaptic GABA_A_ receptors (Yu et al., 2015). It has been hypothesized that the function of this tonic inhibition is to balance the strong excitatory effects of histamine or to increase the spiking precision and, therefore, information processing (Yu et al., 2015; Scammell et al., 2019). Tonic inhibition provided by extrasynaptic GABA_A_ δ receptors can regulate the firing mode of thalamic neurons (Cope et al., 2005) and may destabilize thalamocortical oscillations (Bright et al., 2007).

The fact that histamine/GABA neurons do not make many conventional synaptic contacts and the recent electrophysiological data suggest that GABA most likely acts on various high-affinity extrasynaptic GABA_A_ receptors (Takagi et al., 1986; Yu et al., 2015). Although there are several types of extrasynaptic GABA_A_ receptors expressed in the brain, most of them harbor the δ subunit (Brickley and Mody, 2012). Therefore, we used mice lacking GABA_A_ δ subunits (Gabrd KO) (Mihalek et al., 1999) and pharmacologically manipulated the release of GABA and histamine from the histamine/GABA neurons in order to test whether abolition of tonic GABAergic inhibition modulates the responses to altered histamine/GABA release. We blocked inhibitory Hrh3 autoreceptors on histamine/GABA hypothalamic neurons, which increases transmitter release from these neurons and produces sustained wakefulness (Arrang et al., 1983; Ligneau et al., 1998; Ligneau et al., 2007; Schwartz, 2011; Nieto-Alamilla et al., 2016).

We hypothesized that removal of Hrh3 negative feedback on histamine/GABA neurons will induce release of both histamine and GABA, which will lead to a hypervigilant phenotype in Gabrd KO mice lacking the balancing extrasynaptic GABA_A_ δ receptors. We used locomotor activity assay and electroencephalogram/electromyogram (EEG/EMG) recording to assess the effects of pharmacological treatments. To verify that wake-promoting effect of the Hrh3 antagonist/inverse agonist ciproxifan was due to enhanced histamine release, we pre-treated mice with α-fluoromethylhistidine (αFMH), an irreversible inhibitor of Hdc (Maeyama et al., 1982; Watanabe et al., 1990).

## Methods

### Animals

The principles of the Finnish Act on the Use of Animals for Experimental Purposes were followed, and all protocols were approved by the Animal Experiment Committee of the State Provincial Office of Southern Finland. Animals were group-housed in individually ventilated cages. Access to food pellets and water was assured *ad libitum*. The condition of each mouse was evaluated on a daily basis. Animal rooms were maintained on a 12–12 h light–dark cycle (lights on at 6 a.m.). Temperature and humidity were controlled at 20 ± 1°C and 50 ± 10%, respectively.

Seven 8- to 12-week-old C57BL/6J mice were used for dFISH. In total 150 littermate Gabrd WT and KO female and male mice (Mihalek et al., 1999) derived from heterozygous breedings were used. One group of the mice was used for locomotor activity assay after ciproxifan and then pitolisant treatment, another group was used for locomotor activity assay after ciproxifan treatment and EEG study. The washout period between the tests was at least one week. The mice were 3 to 6 months old at the start of locomotor activity assay and 6 to 7 months at the start of EEG experiment.

### Chemicals

Ciproxifan hydrochloride (AOB 33754; Aobious, Gloucester, MA, USA) and pitolisant hydrochloride (AOB2752; Aobious, Gloucester, MA, USA) were dissolved in saline. Drug doses correspond to free bases of the compounds. Injections were given intraperitoneally (i.p.) and the injection volume was 0.01 ml/g body weight.

Alpha-fluoromethylhistidine hydrochloride (αFMH) was a kind gift from Dr. J. Kollonitch (Merck Sharp & Dohme, Rahway, N.J., USA) and was also dissolved in saline. The drug dose 50 mg/kg corresponds to salt of the compound.

Drugs used in the surgeries were lidocaine (10 mg/ml, Orion Pharma, Finland), carprofen (50 mg/ml, Rimadyl, Pfizer, USA), and buprenorphine (0.3 mg/ml, Temgesic, Reckitt Benckiser, Slough, UK) and isoflurane (induction, 5%; maintenance, 1.8–2.5%; Attane, Piramal Healthcare, Bethlehem, PA, USA).

### Double Fluorescence *In Situ* Hybridization

Mice (n = 7) were transcardially perfused with 4% PFA as described (Abdurakhmanova et al., 2017). Dissected brains were cut on a cryostat, and 25-µm sections were collected on glass slides. Every 10th section from −1.46 to −2.7 Bregma anterior/posterior coordinates (Paxinos and Franklin, 2001) was used for dFISH (n = 5 for *Hdc/Gad67* and n = 4 for *Hdc/Vgat* dFISH; 5–6 sections per animal).

Total RNA was extracted from mouse hypothalamus with Qiagen RNeasy Mini kit (Qiagen, Hilden, Germany) according to manufacturer's protocol. RNA was used for reverse transcription (SuperScript III reverse transcriptase kit) and cDNA synthesis. Fragments of *Hdc* (position 1118–2212 NM_008230.6), *Gad67* (position 1064–2046 NM_008077.4) and *Vgat* (position 866–1818 NM_009508.2) mRNA were amplified from cDNA (DyNAzyme II polymerase kit, Thermo Scientific F-551). The PCR fragments were extracted from 0.7% agarose gel with MinElute gel extraction kit (Qiagen, Hilden, Germany) and cloned to pGEM-T Easy vector (Promega A1360, Fitchburg, WI, USA). DH5α bacteria were transformed with plasmid, and the insert was verified by sequencing. RNA antisense probe for detecting *Hdc* was synthesized from plasmid and labeled with either dinitrophenol (DNP) or fluorescein isothiocyanate (FITC) and probes for detecting *Gad67* and *Vgat* were labeled with digoxigenin (DIG) (RNA labeling kit; Roche, Mannheim, Germany).

Sections were hybridized with *Hdc/Gad67* or *Hdc/Vgat* RNA probes at 65°C in hybridization buffer for 16 to 18 h. Slides were washed in 50% formamide/2.5× SSC (3 × 30 min) and then in 50% formamide/1× SSC (3 × 30 min). Sections were incubated with blocking solution (5% normal sheep serum in PBS-Tween 0.1%) for 1 h at room temperature. Horseradish peroxidase conjugated antibodies against DIG (1:500; Roche, cat 11207733910), DNP (1:200; PerkinElmer cat FP1129) or FITC (1:500; Roche, cat 11426346910) were applied to sections and incubated overnight at 4°C. Probes were detected sequentially: first detection with antibody and tyramide signal amplification (TSA reaction), inactivation of peroxidase with 0.1 M glycine-HCl buffer (pH 2.0, for 10–15 min), incubation with blocking solution and second probe detection. TSA reaction (TAMRA and FAM tyramides) was performed as described (Puttonen, 2017; Puttonen et al., 2018).

### Cell Counting

*Hdc*-positive cells were detected with Zeiss Axio Imager 2 epi-fluorescent microscope and the presence of second mRNA *Gad67* or *Vgat* was assessed. Single or double-positive *Hdc* cells were marked and counted with Stereoinvestigator software (MicroBrightField Inc, Colchester, Vermont, USA).

Representative pictures were taken with Leica TCS SP2 AOBS confocal microscope equipped with a 488-nm argon laser and a 561-nm diode laser. The emission wavelength was set to 500 to 550 nm for FAM and 600 to 670 nm for TAMRA. Stacks of images were taken at 0.32-µm intervals and collected by sequential scanning to reduce the crosstalk between channels.

### Locomotor Activity Assay

Gabrd WT and KO female (n = 38 and n = 41 for WT and KO) and male (n = 39 and n = 32 for WT and KO) mice were treated with saline, ciproxifan 1, 3 or 10 mg/kg (n = 14–22 animals per group) and placed back to the home cage. Thirty minutes after treatment mice were placed in the center of the open field without habituation period. Locomotor activity was tracked in a testing chamber (43.2 × 43.2 × 30.5 cm) equipped with x and y infrared 16-beam arrays for horizontal movements and one 16-beam array for vertical activity (MedAssociates, Georgia, Vermont, USA) for 30 min. Tracks were analyzed with Activity monitor software version 6.02 (MedAssociates). Parameters, such as distance travelled and vertical activity (vertical counts or number of times that the animal rears and vertical time or time spent rearing in seconds), were measured.

For testing the effect of pitolisant on locomotion, Gabrd WT and KO mice (females WT n = 17, KO n = 18; males WT n = 17, KO n = 13) were treated with saline, pitolisant 5 or 15 mg/kg (n = 8–13 animals per group) and placed back to the home cage. Thirty minutes after treatment the mice were placed in the center of an empty plastic cage (40 × 26 × 20 cm) without a habituation period. Locomotor activity of the animals was monitored with a video camera and Ethovision XT 10.1 (Noldus Information Technology, Wageningen, Netherlands) software for 30 min.

Open field tests were performed between 8 and 11 am.

### Surgery

Male mice (Gabrd WT n = 9; KO n = 8) were operated under isoflurane anesthesia (5% and ~2%, induction and maintenance, respectively). Mice received subcutaneous injection of carprofen (5 mg/kg) and infiltration of lidocaine (approximately 50 µl) at the incision site before the start of the procedure. The incision site was disinfected using Betadine (Takeda Oy, Helsinki, Finland) before exposing of the *calvaria*. *Calvaria* was later cleaned from blood, and exposed skull was dried with hydrogen peroxide (3% v/v in saline). The mice were implanted with two gold-coated screws (Surtex gold-plated posts RST-S4, Dentatus, Spanga, Sweden). One recording electrode was implanted to the frontal cortex and the other one to the contralateral parietal cortex for the frontoparietal epidural recording of EEG (**Figure 3A**). EMG electrodes (PFA-coated silver wire, cat. 785500; A-M Systems, WA, USA) were implanted in the neck musculature. To enhance the dental cement adhesion to the *calvaria*, the skull bone was coated with superglue and the electrodes and screws were secured to the scull with dental cement (Candulor, Wangen, Germany). Buprenorphine (0.1 mg/kg) was injected i.p. after the operation to minimize postoperative pain. Mice were left to recover for at least one week before connecting to the EEG/EMG recording system. Weight and general appearance of operated mice were monitored.

### EEG Recording and Analyses

First, mice were habituated to EEG/EMG recording system (attached to contra balanced recording cables) at least for two days. EEG/EMG signals were amplified (gain 10 000) and sampled at 1000 Hz using Spike2 software (version 8.07, Cambridge Electronic Devices, Cambridge, UK).

Baseline 24-h EEG recordings (Gabrd WT n = 9; KO n = 8) were manually scored for artifacts.

EEG signals were down-sampled to 200 Hz, bandpass filtered and signals obtained were Hilbert transformed to extract power-frequency information. Bandpass windows were constructed with fir1 Matlab function as follows: lower cutoff frequencies from 1 to 97.6 Hz and higher cutoff frequencies from 2.6 to 99.2 Hz with 1.4-Hz interval.

For further analyses EEG recordings were binned at 4-s intervals by using median value of the bin. Epochs, which were marked as artifacts were removed from further analyses.

The analysis was performed in Matlab R2018a (Mathworks) using a custom-written code (Cohen, 2014).

The data was then used for plotting EEG power spectrum and further analyses of delta (1–4 Hz), theta (4–8 Hz), alpha (8–12 Hz), sigma (10–15 Hz), beta (12–30 Hz), gamma1 (30–50 Hz) and gamma2 bands (50–100 Hz). For analyses of different band frequencies across bands were averaged and normalized to total power (sum of powers across all bands/per each time point). Data was further binned at 1-h intervals by averaging values within the interval.

Next, the mice (Gabrd WT n = 8; KO n = 7) were treated with either saline or ciproxifan 3 mg/kg and recorded for 4 h. After a washout period (at least one week) the mice were treated with either saline or ciproxifan 10 mg/kg (Gabrd WT n = 9; KO n = 8). Finally, after a washout period (at least two weeks) the mice were pre-treated with irreversible inhibitor of Hdc enzyme, αFMH 50 mg/kg i.p (at 5 pm). and after ~20 h (at 12 pm next day) treated with ciproxifan 10 mg/kg (Gabrd WT n = 8; KO n = 7) and recorded for ~ 5 h. αFMH is a long-lasting histamine synthesis inhibitor, which at the dose 25 mg/kg i.p. blocks Hdc enzyme activity to nearly 25% and decrease brain histamine to 50% of the initial levels after 24 h post-injection (Maeyama et al., 1982).

EEG/EMG signals were recorded during the lights-on period (inactive period for nocturnal mice). EEG recordings were manually scored for vigilance states (wakefulness, NREM, and REM sleep) and artifacts. Scoring was performed on 4-s epochs. Wakefulness was defined as low-amplitude, high-frequency desynchronized EEG signal accompanied by high EMG activity; NREM sleep was defined as high-amplitude, low-frequency signal with low EMG signal; REM sleep was defined as regular low-amplitude, high-frequency signal with absent EMG signal.

Power-frequency analysis of EEG signals was performed as described above. Power of frequencies across bands were averaged and normalized to baseline power (median value across 24-h baseline recording for each analyzed frequency). Further binning at 10-min intervals was made by averaging values within the interval.

### Statistical Analyses

Locomotor activity data was analyzed with two-way ANOVA with Tukey's *post hoc* tests (“car” R package, (Fox and Weisberg, 2018)).

EEG/EMG data (vigilance states and band powers) was analyzed with non-parametric rank-based method, due to violations of assumptions of parametric statistical methods [“nparLD” R package, (Noguchi et al., 2012)]. Post hoc analysis results were corrected with Holm-Bonferroni correction. Detailed statistics for combination of factors (treatment, genotype, time) are included in the **Supplementary Table 1**.

Baseline EEG power spectrum during lights-on and -off times was analyzed with t-test for each frequency. Wake, NREM, and REM relative amount and bout duration were analyzed with *post hoc* t-tests for each time interval (saline vs ciproxifan 3 or 10 mg/kg). Significance threshold for t-tests was p < .05.

Data is represented as mean ± SEM. Statistics were done using R 3.5.0 (R Core Team, 2018).

Significance codes used in the figures:

* *p* ≤ 0.05, ** *p* ≤ 0.01, *** *p* ≤.001 for differences between the genotypes

# *p* ≤ 0.05, ## *p* ≤ 0.01, ### *p* ≤ 0.001 for differences between the treatments

## Results

### Co-Localization of *Gad67* and *Vgat* With *Hdc* mRNA

**Figure 1:**
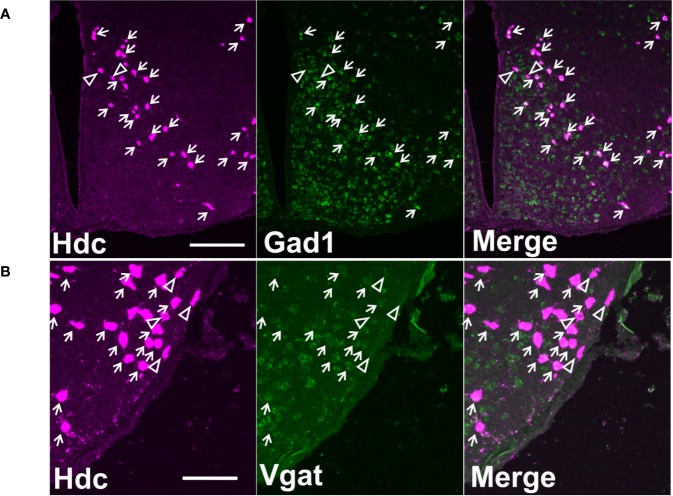
Co-localization of *Hdc* mRNA with *Gad67* or *Vgat* mRNA on maximum intensity projection images. **(A)** A representative image of double fluorescence *in situ* hybridization of *Hdc* and *Gad67* in a coronal section of mouse brain on level −2.30 mm from Bregma. Scale bar is 200 µm. **(B)** A representative image of double fluorescence *in situ* hybridization of *Hdc* and *Vgat* in a coronal section of mouse brain on level −2.30 mm from Bregma. Scale bar is 100 µm. Small arrows indicate double-stained neurons, arrowheads denote cells with only one marker.

Double *in situ* hybridization analyses showed that the majority of *Hdc* positive cells also express *Gad67* (**Figure 1A**) and *Vgat* mRNA (**Figure 1B**). On average, 210 ± 27 (for *Hdc/Gad67* dFISH) and 232 ± 33 (for *Hdc/Vgat* dFISH) *Hdc*-positive cells were counted per animal (mean ± SEM). Average proportion of double-positive cells from the total number of counted Hdc cells were: 99.82 ± 0.12% (n = 5) for *Gad67* and 93.43 ± 0.34% (n = 5) for *Vgat* (values expressed as mean ± SEM).

### Effect of Hrh3 Antagonist/Inverse Agonists on Open-Field Locomotor Activity

**Figure 2:**
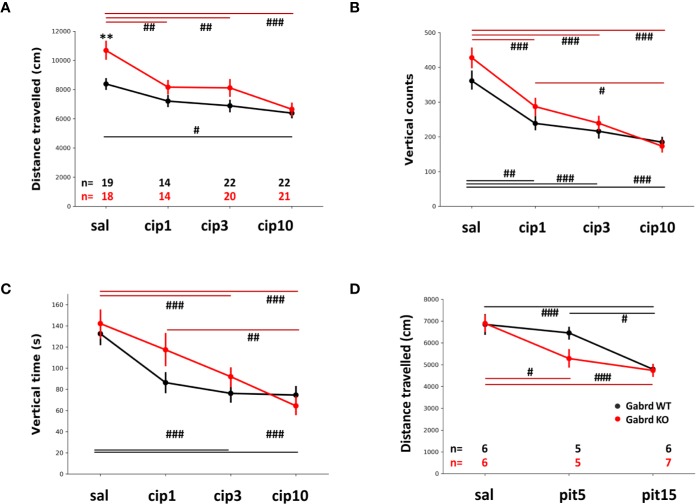
Effect of Hrh3 antagonists/inverse agonists on locomotion during the lights-on period. **(A)** Distance travelled in the open field after treatment with saline or different doses of ciproxifan (1, 3 and 10 mg/kg i.p.). **(B)** Vertical counts (frequency of rearings) in the same open field test. Number of animals is the same as in A. panel. **(C)** Vertical time (time spent rearing) in the same open field test. Number of animals is the same as in A. panel. **(D)** Distance travelled in the open field after treatment with saline or different doses of pitolisant (5 and 15 mg/kg i.p.). ^#^*p* ≤ .05, ^##^*p* ≤ .01, ^###^*p* ≤ .001 for differences between the treatments.

Saline-treated Gabrd KO mice travelled longer distance in the open field than WT animals. Ciproxifan treatment led to decreased locomotor activity in both Gabrd WT and KO groups (treatment effect F(3,134) = 15.65, p = 8.8e-09, **Figure 2A**), but the dose response was different between the two genotypes. In Gabrd KO mice all ciproxifan doses decreased distance traveled compared with saline treatment, while in WT animals only 10 mg/kg of ciproxifan significantly decreased locomotion.

Ciproxifan treatment decreased the number of vertical counts (treatment effect F(3,134) = 37.81, p = 2e-16, **Figure 2B**). In addition, there was an overall effect of genotype and gender (KO mice had more vertical counts than WT mice, genotype effect F(1,134) = 4.16, p = 0.04; males had more vertical counts than females, sex effect F(1,134) = 4.27, p=.04). Vertical counts were similarly decreased in Gabrd WT and KO mice by ciproxifan compared with saline treated mice.

Vertical time was affected by ciproxifan in a similar manner (treatment effect F(3,134) = 22.69, p = 6.1e-12, **Figure 2C**) in both genotypes. There was an overall effect of all ciproxifan doses, but *post hoc* analyses with multiple corrections showed no effect of the lowest ciproxifan dose (WT saline vs ciproxifan 1 mg/kg; KO saline vs ciproxifan 1 mg/kg). Again, there was an overall effect of genotype and gender (KO mice had increased vertical time compared with WT group, genotype effect F(1,134) = 3.85, p = 0.05; and males had increased vertical time compared with females, sex effect F(1,134) = 47.66, p = 1.8e-10).

Pitolisant treatment decreased locomotor activity similarly to ciproxifan (treatment effect F(2,53) = 18.89, p = 6.4e-07, **Figure 2D**). Gabrd KO mice were sensitive to the lowest dose of pitolisant, 5 mg/kg, while there was no effect in WT mice. A high dose of pitolisant 15 mg/kg decreased locomotion in both genotypes compared with saline-treated groups (p = 7.3e-04 and p = 8.7e-04 for WT and KO, *post hoc* tests).

### Baseline EEG Spectral Power in GABA_A_ δ Subunit KO Mice

**Figure 3:**
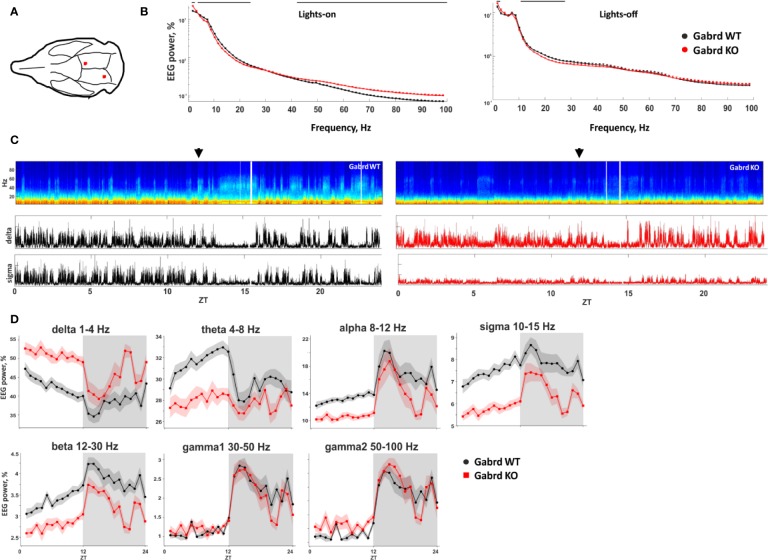
Baseline EEG properties of Gabrd KO mice. **(A)** Schematic representation of EEG electrode placement (red dots). **(B)** EEG power spectrum during inactive (lights-on) and active (lights-off) periods. The black line above shows significant differences between the genotypes (n = 9 and 8 for WT and KO, respectively). **(C)** Representative examples of the power spectrum from 24-h baseline EEG recordings of Gabrd WT and KO mice. Arrows indicate beginning of lights-off time. Lower panels show power of delta and sigma bands. ZT – zeitgeber time. **(D)** Normalized power of frequency bands in Gabrd WT and KO mice (n = 9 and 8 for WT and KO, respectively).

Low and high frequencies were enhanced in KO mice, while mid-range frequencies (5–30 Hz) were suppressed, compared with WT group (**Figures 3B, C**). The differences were most pronounced during inactive (lights-on) period of the animals (**Figure 3B**). Delta power was increased in Gabrd KO mice compared with WT mice (genotype effect χ^2^(1, N = 17), p = 4.04e-05, **Figure 3D**), while theta, alpha, sigma, and beta powers were lower in KO mice (genotype effect χ^2^(1, N = 17), p = 1.18e-03, p = 1.06e-04, p = 1.59e-07, and p = 2.38e-05, respectively, **Figure 3D**). Gamma band powers were similar between the genotypes, although there were significant genotype–time interactions (χ^2^(15, N = 17), p = 1.57e-12, and p = 2.64e-15, respectively, for gamma1 and gamma2 bands, **Figure 3D**).

### Effect of Ciproxifan on Sleep–Wake States

**Figure 4:**
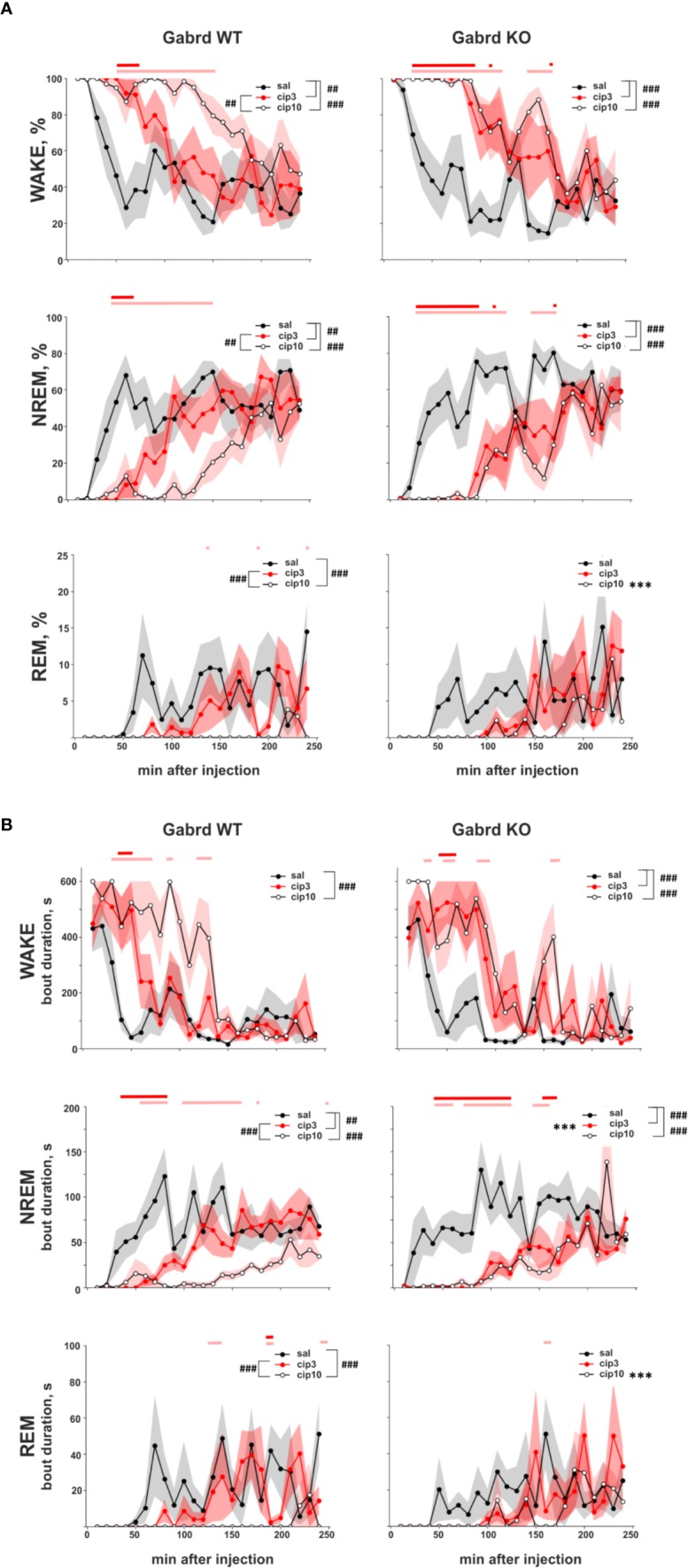
Effect of ciproxifan on vigilance states during the lights-on period. **(A)** Relative amount of Wake, NREM, and REM episodes in Gabrd WT and KO mice during the next 4 h after treatment with saline, ciproxifan 3 or 10 mg/kg. **(B)** Wake, NREM, and REM bout duration in Gabrd WT and KO mice treated with saline, ciproxifan 3 or 10 mg/kg (n = 7, 5, and 5 for WT saline, cip3, and cip10, respectively; n = 6, 4, and 5 for KO saline, cip3, and cip10, respectively). Red and pink bars above show significant differences between the saline and treatment groups for each time interval (red for ciproxifan 3 mg/kg and pink for 10 mg/kg). ***p ≤ .001 for differences between the genotypes. ^##^*p* ≤ .01, ^###^*p* ≤ .001 for differences between the treatments.

Ciproxifan treatment led to sustained wakefulness and decreased relative amount of NREM and REM episodes (treatment effect χ^2^(2, N = 32), p = 7.7e-19, p = 1.2e-22, p = 8.9e-09, **Figure 4A**). Both doses 3 and 10 mg/kg of ciproxifan increased the relative number of wake episodes in Gabrd WT and KO mice compared with corresponding saline treated groups. In WT mice ciproxifan increased the number of wake episodes dose-dependently, while in Gabrd KO mice there was no significant difference between the two doses of ciproxifan. The relative amount of NREM episodes was decreased in both genotypes and again a dose-dependent response was observed in WT mice. Overall, there was a significant effect of treatment on REM sleep (treatment effect χ^2^(2, N = 32), p = 8.9e-09). REM sleep suppression after 3 mg/kg ciproxifan did not reach statistical significance in either genotype. The effect of the higher 10-mg/kg dose of ciproxifan was highly significant in WT mice, but not in Gabrd KO mice. Ciproxifan 3 mg/kg induced sustained wakefulness and NREM sleep suppression in Gabrd KO mice, while Gabrd WT mice returned to the baseline level faster (**Figure 4A**, *post hoc* t-tests).

A similar effect of ciproxifan was found on bout duration. The wake bout duration was increased (treatment effect χ^2^(2, N = 32), p = 2e-14, **Figure 4B**), but NREM and REM bout durations were decreased after the ciproxifan (treatment effect χ^2^(2, N = 32), p = 8.5e-18, p = 8.8e-09, **Figure 4B**). The wake bout duration after 3 mg/kg ciproxifan treatment was significantly increased in Gabrd KO mice, but not in WT mice. The NREM bout duration was decreased by both doses of ciproxifan in both genotypes. However, in WT mice the response was dose dependent, while in Gabrd KO mice there was no difference between the two doses of ciproxifan used. NREM bout duration was shorter in Gabrd KO mice than in WT mice after 3 mg/kg ciproxifan treatment. In addition, NREM bout duration reached the control level later in Gabrd KO mice than in WT (**Figure 4B**, *post hoc* t-tests). The effect of 3 mg/kg ciproxifan on REM bout duration was not significant in either genotype. Ciproxifan 10 mg/kg decreased REM bout duration in the WT group, but not in the Gabrd KO group.

### Effect of Ciproxifan on EEG Spectral Power

**Figure 5:**
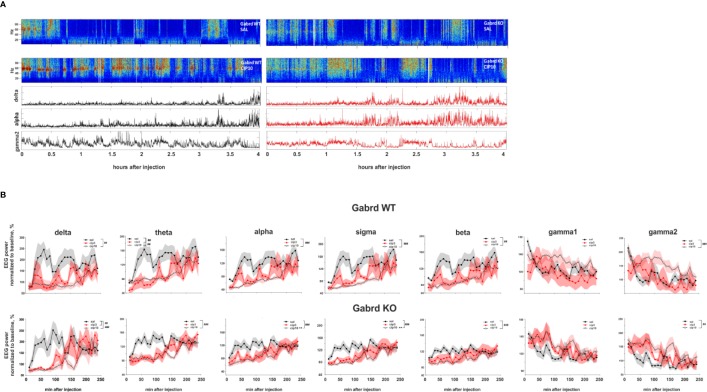
Effect of ciproxifan on EEG power spectrum. **(A)** Example power spectrum from EEG recordings of Gabrd WT and KO mice after treatment with saline or ciproxifan 10 mg/kg. Lower panels show power of delta, alpha, and gamma2 bands in Gabrd WT and KO mice after treatment with ciproxifan 10 mg/kg. **(B)** Normalized power of frequency bands in Gabrd WT and KO mice treated with saline, ciproxifan 3 or 10 mg/kg (n = 7, 5, and 5 for WT saline, cip3, and cip10, respectively; n = 6, 4, and 5 for KO saline, cip3, and cip10, respectively).

Ciproxifan treatment suppressed delta power (treatment effect χ^2^(2, N = 32), p = 6.4e-06, **Figures 5A, B**). While the high dose of ciproxifan reduced delta power in both genotypes, treatment with 3 mg/kg of ciproxifan significantly reduced delta power in Gabrd KO mice, but not in WT mice. Ciproxifan suppressed theta power (treatment effect χ^2^(2, N = 32), p = 1.33e-06, **Figure 5B**). In addition, there was a significant overall effect of genotype (χ^2^(1, N = 32), p = 4.52e-02). For theta frequencies, the effect of 3 mg/kg of ciproxifan was opposite to delta waves: 3 mg/kg of ciproxifan reduced theta power in Gabrd WT mice, but not in Gabrd KO mice.

The high 10 mg/kg dose of ciproxifan suppressed alpha power, while the effect of 3 mg/kg ciproxifan did not reach significance threshold (**Figure 5B**). Furthermore, alpha power of Gabrd KO mice was higher than in WT mice after treatment with the high dose of ciproxifan (**Figures 5A, B**). The effect of ciproxifan on sigma oscillations was similar to alpha waves, sigma power of Gabrd KO mice was higher than in WT mice after treatment with the high dose of ciproxifan (**Figure 5B**). Ciproxifan suppressed beta power (treatment effect χ^2^(2, N = 32), p = 5e-05, **Figure 5B**). Post hoc analyses showed no significant effect after 3 mg/kg ciproxifan in WT and KO. The high dose of ciproxifan suppressed beta power in both Gabrd WT and Gabrd KO mice.

Although there was an overall effect of ciproxifan treatment (χ^2^(2, N = 32), p = 8.38e-03, **Figure 5B**) and ciproxifan treatment seemed to increase gamma1 power, *post hoc* analyses did not reveal any significant effect of ciproxifan doses analyzed separately in Gabrd WT and Gabrd KO mice. The highest dose of ciproxifan increased gamma2 power in both genotypes, while the effect of 3 mg/kg ciproxifan did not reach significance threshold (**Figures 5A, B**).

### Effect of Alpha-FMH Pre-Treatment on Ciproxifan-Induced Wakefulness

**Figure 6:**
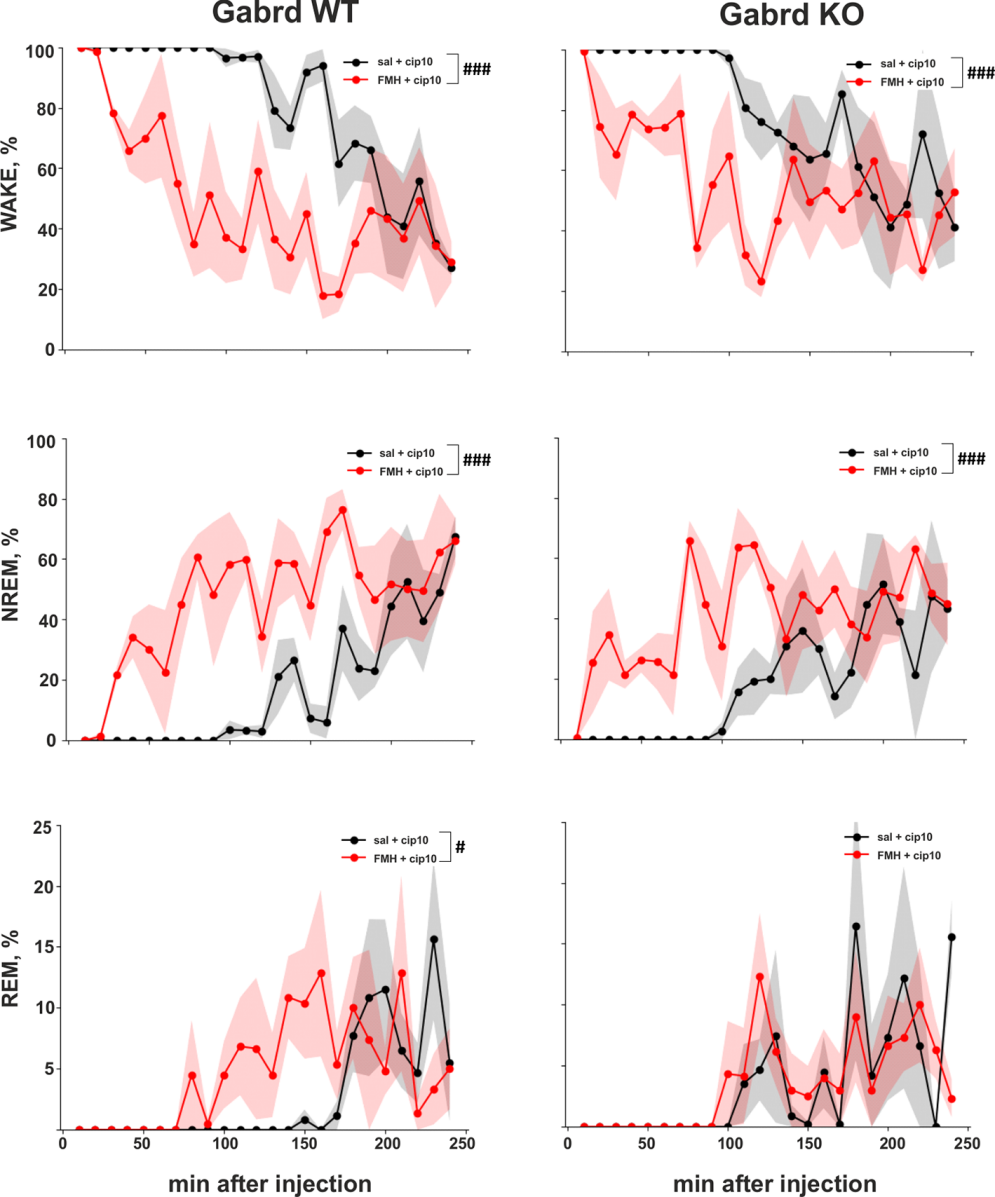
Effect of αFMH pre-treatment on ciproxifan-induced sustained wakefulness. Relative amount of Wake, NREM, and REM episodes in Gabrd WT and KO mice pre-treated with either saline or αFMH and 20 h later treated with ciproxifan 10 mg/kg (n = 4 for WT saline+ciproxifan and αFMH+ ciproxifan; n = 3 and n = 4 for KO saline+ciproxifan and αFMH+ ciproxifan, respectively). ^#^*p* ≤ .05, ^###^*p* ≤ .001 for differences between the treatments.

Pre-treatment with αFMH suppressed ciproxifan (10 mg/kg)-induced sustained wakefulness (treatment effect χ^2^(1, N = 15), p = 7.16e-19, **Figure 6**) and promoted NREM and REM sleep (treatment effects χ^2^(1, N = 15), p = 8.38e-19, p=.03, **Figure 6**). Post hoc analyses showed similar effects of αFMH pre-treatment on the number of wake and NREM sleep episodes in WT and Gabrd KO mice. αFMH pre-treatment rescued REM sleep in WT mice, but in KO mice there was no difference between the two treatments.

## Discussion

Double fluorescence *in situ* hybridization of *Hdc* mRNA with *Gad67* or *Vgat* mRNA showed high co-localization rates, which further supports the hypothesis of vesicular release of GABA from histamine/GABA neurons. The reasons for discrepancy between recently published data (Venner et al., 2019), where *Vgat* mRNA was found only in a few Hdc immune-positive cells, and our study are unclear. Methodological differences (double fluorescence *in situ* hybridization vs fluorescence *in situ* hybridization and immunostaining; *in situ* hybridization technique: hybridization temperature, washing stringency) or regional differences (histaminergic neurons throughout the anterior-posterior axis vs only the ventral subregion of the histaminergic neuron population) may play a role.

Interestingly, while optogenetic stimulation of histaminergic axons in the neocortex and striatum induces direct tonic GABA current (Yu et al., 2015), no such effect was found upon stimulation of histaminergic fibers in ventrolateral preoptic nucleus area (Williams et al., 2014). Given that we found *Gad67* and *Vgat* mRNA in a majority of histamine/GABA neurons it is possible that histamine and GABA release is spatially segregated even within one neuron or release of these neurotransmitters is triggered by different stimulation parameters.

In the present study, we used mice lacking GABA_A_ δ subunits (Gabrd KO) (Mihalek et al., 1999) and Hrh3 antagonists to pharmacologically manipulate the release of GABA and histamine from the histamine/GABA neurons in order to understand whether the tonic GABAergic inhibition would limit the excitatory responses to altered histamine/GABA release. We found that Gabrd KO mice were more sensitive to the lower doses of ciproxifan (1 mg/kg) and pitolisant (5 mg/kg) in locomotor activity assay than WT mice. Also, EEG/EMG experiments revealed higher sensitivity of Gabrd KO mice to the lower dose of ciproxifan (3 mg/kg), showing prolonged sustained wakefulness compared with WT mice, in which this dose only led to a short-lasting effect.

Hrh3 antagonist treatment leads to increased wakefulness, but decreased locomotion and rearing as seen by us here and reported also earlier [(Ligneau et al., 1998; Fox et al., 2003; Ligneau et al., 2007; Vanhanen et al., 2015); but see also (Mohsen et al., 2014)]. It is unclear what the specific mechanism of locomotor regulation by histamine and Hrh3 ligands are, since activation of histamine/GABA neurons with the chemogenetic DREADD (Designer Receptors Exclusively Activated by Designer Drugs) approach leads to increased locomotion (Yu et al., 2015). The decrease in locomotion is unlikely due to direct sedation *via* extrasynaptic inhibition by GABA_A_ δ receptors, since the Gabrd KO mice were more sensitive to the effect. Hrh3 receptors act not only as autoreceptors, providing negative feedback to histamine/GABA neurons, but also as pre- and post-synaptic heteroreceptors on other neuronal populations (Panula et al., 2015; Nieto-Alamilla et al., 2016). For example, Hrh3 presynaptic receptors regulate release of glutamate, GABA, noradrenaline, serotonin, and dopamine (Arrang et al., 1983; Schlicker et al., 1993; Threlfell et al., 2004; Aquino-Miranda et al., 2012; Takei et al., 2017). Therefore, it is possible that the decrease in locomotion was mediated by Hrh3 located on other neurons.

There was a clear difference in baseline EEG properties between Gabrd WT and KO mice, especially during the lights-on (inactive) period. In Gabrd KO mice delta (1–4 Hz) and gamma2 (50–100 Hz) band powers were more prominent compared with those in the WT mice. A previous study found a similar increase of baseline NREM delta power in Gabrd KO mice (Mesbah-Oskui et al., 2014). The authors speculated that such differences would indicate more hyperpolarized resting membrane potential in thalamocortical neurons of Gabrd KO mice due to compensational changes. In the mouse cerebellar granule cells, abolition of GABA_A_ δ receptor-mediated inhibition promotes expression of potassium leak channel TASK-1, providing compensatory baseline hyperpolarization (Brickley et al., 2001), but similar changes in forebrain neurons in the Gabrd KO mice have not been studied.

The waking effect of 3 mg/kg ciproxifan was more pronounced in Gabrd KO mice. Relative amount and bout duration of NREM sleep were suppressed by treatment in a greater extent in Gabrd KO mice. The effect of ciproxifan on power of frequency bands was also different between Gabrd WT and KO mice. For example, delta band of Gabrd KO mice, which was more pronounced in KO mice at the baseline, was significantly suppressed by ciproxifan 3 mg/kg treatment. In contrast, theta band, which had lower baseline power in Gabrd KO mice, was suppressed in WT, but not in Gabrd KO mice. Interestingly, gaboxadol, GABA_A_ δ receptor-preferring hypnotic agonist, has been shown to induce strong EEG delta waves in WT mice, but not in Gabrd KO mice (Winsky-Sommerer et al., 2007), indicating that pharmacological enhancement of extrasynaptic inhibition potentiates the delta EEG frequencies *via* GABA_A_ δ receptors. Therefore, compensatory changes in the Gabrd KO mice might explain their higher baseline delta band activity. The greater suppression of delta band EEG in Gabrd KO mice by Hrh3 antagonists can then be explained by increased histamine release, in the absence of counteracting extrasynaptic GABA_A_ inhibition.

Effects of the higher dose of ciproxifan 10 mg/kg were more evident in WT mice than in Gabrd KO mice. REM sleep was significantly suppressed by the treatment in WT mice, but not in Gabrd KO mice. Alpha and sigma bands were more suppressed by the treatment in WT mice than in KO mice.

The wake-promoting effect of the Hrh3 antagonist ciproxifan, as analyzed *via* EEG recordings, was attenuated by αFMH treatment, leading to depletion of histamine, with both mouse lines showing similar responses, suggesting that histamine is responsible for the observed differences between the genotypes.

The rodent EEG signal mainly reflects the activity of the cerebral cortex and hippocampus, due to the location of electrodes, cytoarchitecture and synchronized neuronal activity of these brain structures, and the thalamus, which tightly regulates cortical activity (Buzsáki et al., 2012). It has been shown that GABA from the histamine/GABA neurons induces inhibitory tonic currents in cortical pyramidal neurons (Yu et al., 2015), which strongly contribute to generation of synchronized activity detected by EEG. Sparse histamine-immunoreactive fibers are found in the cortex and hippocampus (Panula et al., 1989) suggesting paracrine-like volume transmission of released histamine in these brain areas. GABA_A_ δ receptors are also present in the cortex and hippocampus, although their density is highest in the thalamus and cerebellum (Wisden et al., 1992). The hypervigilant EEG phenotype in Gabrd KO mice after ciproxifan treatment is then most probably due to histamine-induced over-excitation and lack of GABA_A_ δ receptor-mediated inhibition in the cortex, hippocampus, and thalamus. The cortical pyramidal neurons might be the main contributor in the observed effect as they are the closest to the recording electrodes. Neuronal type affected by GABA released from histamine/GABA neurons remains yet to be established in other brain regions.

It should be noted that in addition to GABA_A_ δ subunit-containing receptors there are also other GABA receptors providing tonic extrasynaptic inhibition including GABA_A_ receptors composed of α5βγ2 subunits (Brickley and Mody, 2012) and GABA_B_ receptors. GABA_B_ receptors are not only able to induce extrasynaptic currents (Scanziani, 2000), but they also enhance tonic GABA_A_ currents (Connelly et al., 2013). Therefore, the magnitude of the wake-promoting effect of Hrh3 inverse agonists might be even larger in the absence of also the other forms of high-affinity extrasynaptic GABA_A_ receptors (Chandra et al., 2010).

We should point out technical limitations of the study that require careful interpretation of the results. First, there are compensatory changes due to global knock-out of GABA_A_ δ subunit gene. Alterations in assembly of other GABA_A_ receptor subunits have been found in Gabrd KO mice (Korpi et al., 2002). Secondly, we used a pharmacological approach to block Hrh3 autoreceptors and induce release of neurotransmitters from histamine/GABA neurons. The treatment of mice with the Hrh3 antagonist/inverse agonist ciproxifan indeed led to sustained wakefulness, which was blocked with αFMH pre-treatment, suggesting that histamine is essential for wakefulness. Nevertheless, Hrh3 receptors act not only as autoreceptors, providing negative feedback to histamine/GABA neurons, but also as pre- and post-synaptic receptors on other neuronal populations (Panula et al., 2015; Nieto-Alamilla et al., 2016). Non-pharmacological approaches should also be examined, since unlike the Hrh3 antagonist treatments, selective activation of histaminergic neurons leads to hyperlocomotion (Yu et al., 2015).

To summarize, we showed increased sensitivity of Gabrd KO mice to the locomotion suppression effects of the Hrh3 antagonists/inverse agonists ciproxifan and pitolisant. Treatment of Gabrd KO mice with the lower dose of ciproxifan led to sustained wakefulness and inhibition of low frequency delta oscillations in a greater extent than that of WT mice, consistent with our initial hypothesis. Treatment with the higher dose of ciproxifan led to more pronounced effects in WT than in KO mice, reflecting, possibly, a ceiling effect of a ligand or compensational changes in KO mice. Another possibility is that the different isoforms of Hrh3 (Drutel et al., 2001; Panula et al., 2015) on different domains of the circuits involved are recruited at different ligand concentrations.

## Data Availability Statement

The datasets generated for this study are available on request to the corresponding author.

## Ethics Statement

The animal study was reviewed and approved by Animal Experiment Committee of the State Provincial Office of Southern Finland.

## Author Contributions

SA, MG, A-ML, EK, and PP designed the experiments. SA, MG, and JK performed the experiments. SA analyzed the data and wrote the manuscript. SA, MG, A-ML, EK, and PP revised the manuscript.

## Funding

The study was supported by the Academy of Finland (253416, 1317399), Sigrid Juselius Foundation, Magnus Ehrnrooth's foundation and Finska Läkaresällskapet.

## Conflict of Interest

The authors declare that the research was conducted in the absence of any commercial or financial relationships that could be construed as a potential conflict of interest.

## References

[B1] Abdurakhmanova S. Chary K. Kettunen M. Sierra A. Panula P. (2017). Behavioral and stereological characterization of Hdc KO mice: Relation to Tourette syndrome. J. Comp. Neurol. 525, 3476–3487. 10.1002/cne.24279 28681514

[B2] Airaksinen M. S. Alanen S. Szabat E. Visser T. J. Panula P. (1992). Multiple neurotransmitters in the tuberomammillary nucleus: comparison of rat, mouse, and guinea pig. J. Comp. Neurol. 323, 103–116. 10.1002/cne.903230109 1385490

[B3] Aquino-Miranda G. Osorio-Espinoza A. Escamilla-Sanchez J. Gonzalez-Pantoja R. Ortiz J. Arias-Montano J. A. (2012). Histamine H(3) receptors modulate depolarization-evoked [(3)H]-noradrenaline release from rat olfactory bulb slices. Neuropharmacology 62, 1127–1133. 10.1016/j.neuropharm.2011.11.004 22115898

[B4] Arrang J. M. Garbarg M. Schwartz J. C. (1983). Auto-inhibition of brain histamine release mediated by a novel class (H3) of histamine receptor. Nature 302, 832–837. 10.1038/302832a0 6188956

[B5] Brickley S. G. Mody I. (2012). Extrasynaptic GABA(A) receptors: their function in the CNS and implications for disease. Neuron 73, 23–34. 10.1016/j.neuron.2011.12.012 22243744 PMC3399243

[B6] Brickley S. G. Revilla V. Cull-Candy S. G. Wisden W. Farrant M. (2001). Adaptive regulation of neuronal excitability by a voltage-independent potassium conductance. Nature 409, 88. 10.1038/35051086 11343119

[B7] Bright D. P. Aller M. I. Brickley S. G. (2007). Synaptic release generates a tonic GABA(A) receptor-mediated conductance that modulates burst precision in thalamic relay neurons. J. Neurosci. 27, 2560–2569. 10.1523/JNEUROSCI.5100-06.2007 17344393 PMC6672513

[B8] Brown R. E. Stevens D. R. Haas H. L. (2001). The physiology of brain histamine. Prog. Neurobiol. 63, 637–672. 10.1016/S0301-0082(00)00039-3 11164999

[B9] Buzsáki G. Anastassiou C. A. Koch C. (2012). The origin of extracellular fields and currents–EEG, ECoG, LFP and spikes. Nat. Rev. Neurosci. 13, 407–420. 10.1038/nrn3241 22595786 PMC4907333

[B10] Chandra D. Halonen L. M. Linden A. M. Procaccini C. Hellsten K. Homanics G. E. (2010). Prototypic GABA(A) receptor agonist muscimol acts preferentially through forebrain high-affinity binding sites. Neuropsychopharmacol. : Off. Publ. Am. Coll. Neuropsychopharmacol. 35, 999–1007. 10.1038/npp.2009.203 PMC2823376 20032968

[B11] Cohen M. X. (2014). Analyzing neural time series data: theory and practice (Cambridge, MA, UK:MIT press).

[B12] Connelly W. M. Fyson S. J. Errington A. C. McCafferty C. P. Cope D. W. Di Giovanni G. (2013). GABAB Receptors Regulate Extrasynaptic GABAA Receptors. J. Neurosci. 33, 3780–3785. 10.1523/JNEUROSCI.4989-12.2013 23447590 PMC3601669

[B13] Cope D. W. Hughes S. W. Crunelli V. (2005). GABAA Receptor-Mediated Tonic Inhibition in Thalamic Neurons. J. Neurosci. 25, 11553–11563. 10.1523/JNEUROSCI.3362-05.2005 16354913 PMC6726040

[B14] Drutel G. Peitsaro N. Karlstedt K. Wieland K. Smit M. J. Timmerman H. (2001). Identification of rat H3 receptor isoforms with different brain expression and signaling properties. Mol. Pharmacol. 59 (1), 1–8. 11125017

[B15] Fox J. Weisberg S. (2018). An R companion to applied regression (Thousand Oaks, CA, USA: Sage Publications).

[B16] Fox G. B. Pan J. B. Radek R. J. Lewis A. M. Bitner R. S. Esbenshade T. A. (2003). Two novel and selective nonimidazole H3 receptor antagonists A-304121 and A-317920: II. In vivo behavioral and neurophysiological characterization. J. Pharmacol. Exp. Ther. 305, 897–908. 10.1124/jpet.102.047241 12606600

[B17] Haas H. Panula P. (2003). The role of histamine and the tuberomamillary nucleus in the nervous system. Nat. Rev. Neurosci. 4, 121–130. 10.1038/nrn1034 12563283

[B18] Korpi E. R. Mihalek R. M. Sinkkonen S. T. Hauer B. Hevers W. Homanics G. E. (2002). Altered receptor subtypes in the forebrain of GABA(A) receptor delta subunit-deficient mice: recruitment of gamma 2 subunits. Neuroscience 109, 733–743. 10.1016/S0306-4522(01)00527-9 11927155

[B19] Kukko-Lukjanov T. K. Panula P. (2003). Subcellular distribution of histamine, GABA and galanin in tuberomamillary neurons in vitro. J. Chem. Neuroanat. 25, 279–292. 10.1016/S0891-0618(03)00043-7 12842273

[B20] Ligneau X. Lin J. Vanni-Mercier G. Jouvet M. Muir J. L. Ganellin C. R. (1998). Neurochemical and behavioral effects of ciproxifan, a potent histamine H3-receptor antagonist. J. Pharmacol. Exp. Ther. 287, 658–666. 9808693

[B21] Ligneau X. Perrin D. Landais L. Camelin J. C. Calmels T. P. Berrebi-Bertrand I. (2007). BF2.649 [1-{3-[3-(4-Chlorophenyl)propoxy]propyl}piperidine, hydrochloride], a nonimidazole inverse agonist/antagonist at the human histamine H3 receptor: Preclinical pharmacology. J. Pharmacol. Exp. Ther. 320, 365–375. 10.1124/jpet.106.111039 17005916

[B22] Maeyama K. Watanabe T. Taguchi Y. Yamatodani A. Wada H. (1982). Effect of α-fluoromethylhistidine, a suicide inhibitor of histidine decarboxylase, on histamine levels in mouse tissues. Biochem. Pharmacol. 31, 2367–2370. 10.1016/0006-2952(82)90531-7 7126249

[B23] Mesbah-Oskui L. Orser B. A. Horner R. L. (2014). Thalamic δ-subunit containing GABAA receptors promote electrocortical signatures of deep non-REM sleep but do not mediate the effects of etomidate at the thalamus in vivo. J. Neurosci. 34, 12253–12266. 10.1523/JNEUROSCI.0618-14.2014 25209268 PMC6615504

[B24] Mihalek R. M. Banerjee P. K. Korpi E. R. Quinlan J. J. Firestone L. L. Mi Z.-P. (1999). Attenuated sensitivity to neuroactive steroids in γ-aminobutyrate type A receptor delta subunit knockout mice. Proc. Natl. Acad. Sci. 96, 12905–12910. 10.1073/pnas.96.22.12905 10536021 PMC23157

[B25] Mohsen A. Yoshikawa T. Miura Y. Nakamura T. Naganuma F. Shibuya K. (2014). Mechanism of the histamine H(3) receptor-mediated increase in exploratory locomotor activity and anxiety-like behaviours in mice. Neuropharmacology 81, 188–194. 10.1016/j.neuropharm.2014.02.003 24530460

[B26] Nieto-Alamilla G. Marquez-Gomez R. Garcia-Galvez A. M. Morales-Figueroa G. E. Arias-Montano J. A. (2016). The Histamine H3 Receptor: Structure, Pharmacology, and Function. Mol. Pharmacol. 90, 649–673. 10.1124/mol.116.104752 27563055

[B27] Noguchi K. Gel Y. R. Brunner E. Konietschke F. (2012). nparLD: an R software package for the nonparametric analysis of longitudinal data in factorial experiments. J. Stat. Softw. 50, 1–23. 10.18637/jss.v050.i12 25317082

[B28] Panula P. Nuutinen S. (2013). The histaminergic network in the brain: basic organization and role in disease. Nat. Rev. Neurosci. 14, 472–487. 10.1038/nrn3526 23783198

[B29] Panula P. Pirvola U. Auvinen S. Airaksinen M. S. (1989). Histamine-immunoreactive nerve fibers in the rat brain. Neuroscience 28, 585–610. 10.1016/0306-4522(89)90007-9 2710333

[B30] Panula P. Chazot P. L. Cowart M. Gutzmer R. Leurs R. Liu W. L. (2015). International union of basic and clinical pharmacology. XCVIII. Histamine receptors. Pharmacol. Rev. 67, 601–655. 10.1124/pr.114.010249 26084539 PMC4485016

[B31] Paxinos K. Franklin G. (2001). The mouse brain in stereotaxic coordinates. San Diego: Acad. Press 200, 65–69.

[B32] Puttonen H. A. J. Sundvik M. Semenova S. Shirai Y. Chen Y. C. Panula P. (2018). Knockout of histamine receptor H3 alters adaptation to sudden darkness and monoamine levels in the zebrafish. Acta Physiol. (Oxf) 222. 10.1111/apha.12981 29044927

[B33] Puttonen H. (2017). Neuropharmacological Properties of the Histaminergic System in the Zebrafish. Dissertationes Scholae Doctoralis Ad Sanitatem Investigandam Universitatis Helsinkiensis.

[B34] Rozov S. V. Zant J. C. Karlstedt K. Porkka-Heiskanen T. Panula P. (2014). Periodic properties of the histaminergic system of the mouse brain. Eur. J. Neurosci. 39, 218–228. 10.1111/ejn.12397 24438489

[B35] Saito Y. C. Maejima T. Nishitani M. Hasegawa E. Yanagawa Y. Mieda M. (2018). Monoamines Inhibit GABAergic Neurons in Ventrolateral Preoptic Area That Make Direct Synaptic Connections to Hypothalamic Arousal Neurons. J. Neurosci. 38, 6366–6378. 10.1523/JNEUROSCI.2835-17.2018 29915137 PMC6596100

[B36] Scammell T. E. Jackson A. C. Franks N. P. Wisden W. Dauvilliers Y. (2019). Histamine: neural circuits and new medications. Sleep 42, zsy183. 10.1093/sleep/zsy183 PMC6335869 30239935

[B37] Scanziani M. (2000). GABA spillover activates postsynaptic GABA(B) receptors to control rhythmic hippocampal activity. Neuron 25, 673–681. 10.1016/S0896-6273(00)81069-7 10774734

[B38] Schlicker E. Fink K. Detzner M. Gothert M. (1993). Histamine inhibits dopamine release in the mouse striatum via presynaptic H3 receptors. J. Neural Transm Gen. Sect 93, 1–10. 10.1007/BF01244933 8396945

[B39] Schwartz J.-C. (2011). The histamine H3 receptor: from discovery to clinical trials with pitolisant. Br. J. Pharmacol. 163, 713–721. 10.1111/j.1476-5381.2011.01286.x 21615387 PMC3111674

[B40] Sundvik M. Panula P. (2012). Organization of the histaminergic system in adult zebrafish (Danio rerio) brain: neuron number, location, and cotransmitters. J. Comp. Neurol. 520, 3827–3845. 10.1002/cne.23126 22522821

[B41] Takagi H. Morishima Y. Matsuyama T. Hayashi H. Watanabe T. Wada H. (1986). Histaminergic axons in the neostriatum and cerebral cortex of the rat: a correlated light and electron microscopic immunocytochemical study using histidine decarboxylase as a marker. Brain Res. 364, 114–123. 10.1016/0006-8993(86)90992-3 3004646

[B42] Takeda N. Inagaki S. Shiosaka S. Taguchi Y. Oertel W. H. Tohyama M. (1984). Immunohistochemical evidence for the coexistence of histidine decarboxylase-like and glutamate decarboxylase-like immunoreactivities in nerve cells of the magnocellular nucleus of the posterior hypothalamus of rats. Proc. Natl. Acad. Sci. U. S. A 81, 7647–7650. 10.1073/pnas.81.23.7647 6594708 PMC392205

[B43] Takei H. Yamamoto K. Bae Y.-C. Shirakawa T. Kobayashi M. (2017). Histamine H3 Heteroreceptors Suppress Glutamatergic and GABAergic Synaptic Transmission in the Rat Insular Cortex. Front. Neural Circuits 11. 10.3389/fncir.2017.00085 PMC5684127 29170631

[B44] Threlfell S. Cragg S. J. Kallo I. Turi G. F. Coen C. W. Greenfield S. A. (2004). Histamine H3 receptors inhibit serotonin release in substantia nigra pars reticulata. J. Neurosci. 24, 8704–8710. 10.1523/JNEUROSCI.2690-04.2004 15470136 PMC6729965

[B45] Tritsch N. X. Ding J. B. Sabatini B. L. (2012). Dopaminergic neurons inhibit striatal output through non-canonical release of GABA. Nature 490, 262–266. 10.1038/nature11466 23034651 PMC3944587

[B46] Trottier S. Chotard C. Traiffort E. Unmehopa U. Fisser B. Swaab D. F. (2002). Co-localization of histamine with GABA but not with galanin in the human tuberomamillary nucleus. Brain Res. 939, 52–64. 10.1016/S0006-8993(02)02546-5 12020851

[B47] Vanhanen J. Kinnunen M. Nuutinen S. Panula P. (2015). Histamine H3 receptor antagonist JNJ-39220675 modulates locomotor responses but not place conditioning by dopaminergic drugs. Psychopharmacol. (Berl) 232, 1143–1153. 10.1007/s00213-014-3751-7 25308376

[B48] Venner A. Mochizuki T. De Luca R. Anaclet C. Scammell T. E. Saper C. B. (2019). Reassessing the Role of Histaminergic Tuberomammillary Neurons in Arousal Control. J. Neurosci. 39, 8929–8939. 10.1523/JNEUROSCI.1032-19.2019 31548232 PMC6832676

[B49] Watanabe T. Yamatodani A. Maeyama K. Wada H. (1990). Pharmacology of alpha-fluoromethylhistidine, a specific inhibitor of histidine decarboxylase. Trends Pharmacol. Sci. 11, 363–367. 10.1016/0165-6147(90)90181-7 2238092

[B50] Williams R. H. Chee M. J. S. Kroeger D. Ferrari L. L. Maratos-Flier E. Scammell T. E. (2014). Optogenetic-mediated release of histamine reveals distal and autoregulatory mechanisms for controlling arousal. J. Neurosci. 34, 6023–6029. 10.1523/JNEUROSCI.4838-13.2014 24760861 PMC3996219

[B51] Winsky-Sommerer R. Vyazovskiy V. V. Homanics G. E. Tobler I. (2007). The EEG effects of THIP (Gaboxadol) on sleep and waking are mediated by the GABAAδ-subunit-containing receptors. Eur. J. Neurosci. 25, 1893–1899. 10.1111/j.1460-9568.2007.05455.x 17408425

[B52] Wisden W. Laurie D. Monyer H. Seeburg P. (1992). The distribution of 13 GABAA receptor subunit mRNAs in the rat brain. I. Telencephalon, diencephalon, mesencephalon. J. Neurosci. 12, 1040–1062. 10.1523/JNEUROSCI.12-03-01040.1992 1312131 PMC6576059

[B53] Yu X. Ye Z. Houston C. M. Zecharia A. Y. Ma Y. Zhang Z. (2015). Wakefulness Is Governed by GABA and Histamine Cotransmission. Neuron 87, 164–178. 10.1016/j.neuron.2015.06.003 26094607 PMC4509551

[B54] Zant J. C. Rozov S. Wigren H. K. Panula P. Porkka-Heiskanen T. (2012). Histamine release in the basal forebrain mediates cortical activation through cholinergic neurons. J. Neurosci. 32, 13244–13254. 10.1523/JNEUROSCI.5933-11.2012 22993440 PMC6621481

